# Obesity as a Part of Polycysric Ovary Syndrome (PCOS)—A Review of Pathophysiology and Comprehensive Therapeutic Strategies

**DOI:** 10.3390/jcm14165642

**Published:** 2025-08-09

**Authors:** Jovan Bila, Jelena Dotlic, Mladen Andjic, Katarina Ivanovic, Jelena Micic, Lidija Tulic, Miljan Pupovac, Jelena Stojnic, Ivana Vukovic, Stefan Ivanovic

**Affiliations:** 1 Clinic of Obstetrics and Gynecology, University Clinical Center of Serbia, 11000 Belgrade, Serbia; 2 Faculty of Medicine, University of Belgrade, 11000 Belgrade, Serbia; 3 The Obstetrics and Gynecology Clinic Narodni Front, 11000 Belgrade, Serbia

**Keywords:** PCOS, obesity, insulin resistance, GLP-1 agonists, inositols

## Abstract

**Background/Objectives:** Polycystic Ovary Syndrome (PCOS), as a multifactorial chronic disease, can cause heterogeneous metabolic, physical, and psychological disorders as well as infertility in both obese and non-obese patients. Therefore, this review aimed to present differences in pathophysiology, clinical presentation, and therapy in obese and non-obese patients with PCOS. **Methods:** A non-systematic review was conducted by searching papers published in English from 2010 to 2024 in MEDLINE. **Results:** Obesity in PCOS significantly contributes to IR and worsens metabolic dysfunction. Lifestyle modifications, including a balanced diet and regular exercise, are the first line of treatment. Pharmacological therapies, such as metformin, GLP-1 receptor agonists, myoinositol, and resveratrol, are used to improve insulin sensitivity, regulate the hormonal milieu, and reduce hyperandrogenism. Metformin is widely used to improve glucose metabolism and reduce androgen levels, while myoinositol is effective in promoting ovarian function. GLP-1 receptor agonists and resveratrol improve metabolic and reproductive outcomes. For patients with severe obesity, bariatric surgery offers substantial improvements in body composition, metabolic function, and fertility. Combination therapies, such as metformin and GLP-1 receptor agonists, provide comprehensive treatment for both reproductive and metabolic aspects of PCOS. **Conclusions:** The first-line treatment for PCOS is a lifestyle-modifying strategy. PCOS patients with insulin resistance and obesity would mostly benefit from combination therapy with metformin and GLP-1 receptor agonists.

## 1. Introduction

Polycystic ovary syndrome (PCOS) is a reproductive and metabolic disorder in women, typically characterized by chronic oligo-ovulation or anovulation, hyperandrogenism, and/or metabolic disorders. These symptoms, as well as infertility issues caused by PCOS, are one of the major reasons young women seek health care. PCOS is usually diagnosed in the reproductive period between the 2nd and 3rd decade of life. Its global prevalence ranges between 5 and 20% and has been on the rise recently. The worldwide prevalence of PCOS doubled during the past three decades, particularly in the 15–19 and 45–49 age groups, making it one of the most common gynecologic and endocrinologic disorders [1,2].

PCOS can present as an isolated disorder in petite young women, without metabolic syndrome. However, it is frequently related to metabolic abnormalities, such as weight gain and obesity, deregulated glucose tolerance, insulin resistance (IR), and disturbance of lipid profile. Previous studies showed that approximately 50% of patients with PCOS are obese [3,4,5].

In recent decades, obesity has developed into a global public health problem. According to data from the year 2022, approximately 44% of women worldwide were overweight, and up to 10% were obese [3,4,5]. Its prevalence is increasing in all age groups, regardless of ethnicity or socioeconomic status. Obesity by itself significantly increases risks for numerous illnesses such as type 2 diabetes mellitus, hypertension, and other cardiovascular diseases, non-alcoholic fatty liver, certain cancers, musculoskeletal illnesses, chronic kidney disease, obstructive sleep apnea, mental disorders, as well as reproductive system disorders, including infertility. However, specific mechanisms by which obesity can cause reproductive disorders remain unclear [3].

The substantial overlap between PCOS features and metabolic syndrome proposes that impairment of adipocyte function could be one of the main contributing factors of numerous metabolic complications registered in women with PCOS. Weight gain and obesity often pave the way to anovulatory cycles, hirsutism, and menstrual irregularity [4]. IR is the root of the increased adipogenesis and lipogenesis, where the obesity additionally aggravates the hyperandrogenism by exaggerating IR. Earlier studies have shown that visceral adiposity, compared to overall adiposity, is associated with overexpressed IR and increased morbidity [5]. In PCOS patients, visceral fat amount contributes more than subcutaneous fat to the IR due to the fact that lipolytic response to catecholamines is better increased in visceral and decreased in subcutaneous fat [6]. Visceral fat contains abundant beta-adrenergic receptors with a high lipolytic activity. Therefore, excess visceral fat leads to bigger free fatty acid delivery to the liver, thereby disturbing insulin clearance and function. Adipose tissue expresses a variety of enzymes for interconversion, activation, and/or inactivation of steroid hormones, influencing their free fraction and action [7].

Therefore, due to the significant prevalence, diverse etiology, and its connection to IR and metabolic syndrome, PCOS is now regarded as a general health disorder in women instead of just an issue with reproductive or irregular menstrual cycles. In this narrative review, we aimed to present differences in pathophysiology and clinical presentation in obese and non-obese patients with the aim of giving more insights into contemporary treatment options for PCOS accompanied by obesity and metabolic syndrome.

## 2. Materials and Methods

In this narrative review article, the authors investigated the data available in the literature regarding the pathophysiology of PCOS and its current treatment strategies in obese and non-obese patients with PCOS. The literature search of MEDLINE for the years 2010–2024 was performed, using keywords, alone or in combinations, such as “polycystic ovarian syndrome”, “obesity”, “adipose tissue”, “insulin resistance”, “metabolic syndrome”, “pathogenesis”, “infertility”, “life style”, “diet”, “physical activity”, “metformin”, and “treatment”. Peer-reviewed accessible full-text publications, written in English, were included in this review. The authors searched all observational clinical and epidemiological studies, experimental studies, as well as systematic reviews and meta-analyses. We also analyzed manuscripts according to the number of citations of each article. We opted to omit from this study the articles that were never cited before. Titles and abstracts of retrieved studies were screened autonomously by two study authors to determine those that possibly met the aims of this review. The complete text of these potentially suitable articles was downloaded and autonomously evaluated for eligibility by two other team members. Duplicate publications were excluded from this study. Any disagreement between them over the eligibility of particular articles was resolved through discussion with a third collaborator. Two authors autonomously extracted data from manuscripts. In the case of disagreement, issues were registered and resolved through discussion with a third collaborator. The results of the research have been divided into different sections and subsections to illustrate what has been reported on the topic of the discussion.

For the purpose of this study, overweight and obesity were defined as BMI > 25 kg/m^2^ and BMI > 30 kg/m^2^, respectively, including abnormal or excessive fat accumulation that is associated with increased health risk. Obesity is characterized by multifactorially caused increased adiposity, with or without abnormal distribution or function of adipose tissue. Preclinical obesity is a state of excess adiposity with preserved function of tissues and organs and a varying, but generally increased, risk of developing clinical obesity and several other non-communicable diseases. Clinical obesity is a chronic, systemic illness linked with alterations in the function of tissues, organs, and the entire organism due to increased adiposity [8].

This paper has some limitations. Firstly, only free full-text English language manuscripts were included. Secondly, due to the heterogeneous nature of this review, only a narrative synthesis was possible. Included studies may have diverse quality, study design, and outcomes assessed.

## 3. Results

**Figure 1:**
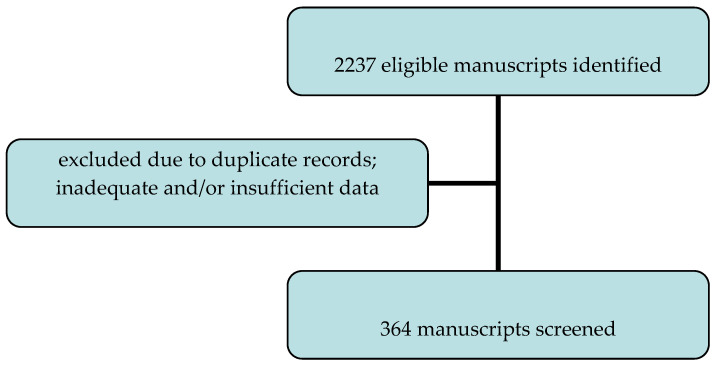
Identification of studies in available databases.

The literature search identified more than 6500 studies regarding obesity issues in patients with PCOS, out of which 2237 were published in the past 15 years in English and are freely available. After thorough analysis according to study inclusion criteria, the data from 71 studies were included in this review (Figure 1).

### 3.1. PCOS Clinical Aspects

According to newest guidelines for the evaluation and treatment of PCOS, which endorses the previous Rotterdam criteria and the Androgen Excess Society guidelines, the diagnosis of PCOS is based on the presence of at least two of three clinical features—clinical or biochemical hyperandrogenism, ovulatory dysfunction (oligo-amenorrhea) or polycystic ovarian morphology (>20 follicles and/or ovarian volume >10 mL) [9]. The main change in the diagnostic guidelines from 2023 is focusing on individual diagnostic criteria with reference to lifestyle, education, emotional well-being, and quality of life [1]. Also, according to the International Consortium guidelines, PCOS diagnosis can be made at least two years after menarche with exclusion of other diseases that could be the cause of hyperandrogenism, hyperprolactinemia, and abnormal thyroid function [10].

The Rotterdam criteria defined different phenotypes of the syndrome. Phenotype A is presented with hyperandrogenism, ovulatory dysfunction, and polycystic ovarian ultrasound morphology. Phenotype B is characterized by hyperandrogenism and ovulatory dysfunction. Women with phenotypes A and B usually also have menstrual dysfunction, increased risk for metabolic syndrome (increased insulin secretion and insulin resistance), dyslipidemia, hepatic steatosis, and a higher incidence of obesity. Ovulatory PCOS with hyperandrogenism and polycystic ovarian ultrasound morphology (Phenotype C) is characterized by slightly increased serum insulin, atherogenic lipids, and androgen levels, and high hirsutism scores. Interestingly, it is commonly found among patients with higher socioeconomic status, likely due to their lifestyle and eating habits. On the other hand, phenotype D with androgen levels in the referral range has the lowest risk of metabolic disorders [10,11].

In order to better predict metabolic and reproductive outcomes according to clinical experience and pathophysiology of PCOS, a division of PCOS into two major subtypes, reproductive and metabolic, was suggested. In the case of reproductive PCOS, luteinizing hormone (LH) and sex hormone binding globulin (SHBG) levels are high, while insulin levels are normal, and body mass index (BMI) is in the referral range or decreased. Metabolic PCOS is linked to high BMI, glucose, and insulin levels, while LH and SHBG levels are decreased [12].

While the Rotterdam criteria remain essential for diagnosing the syndrome and distinguishing phenotypes A–D, they occasionally cannot adequately reflect the heterogeneity in metabolic dysfunction between lean and obese patients with PCOS.

### 3.2. Molecular Basis of PCOS Development

The molecular pathogenesis of PCOS remains incompletely understood, but it is hypothesized to follow a ‘two-hit’ model, where congenital predispositions manifest clinically after postnatal triggers [13]. Functional ovarian hyperandrogenism (FOH) is the root of most PCOS cases, caused by deregulation of androgen secretion. It’s a gonadotropin-dependent disorder, where the expression of the steroidogenic enzymes is dependent on luteinizing hormone (LH) stimulation. Typical FOH is characterized by ovarian 17-hydroxyprogesterone (17-OH-Pg) hyper-responsivity to gonadotropin stimulation, marked by deregulated steroidogenesis, particularly at the level of cytochrome P450c17, which is the main enzyme in the process of androgen synthesis in the ovaries and the adrenal glands [13,14]. The concentrations of androstenedione in theca cells of women with PCOS are 20 times higher than in healthy women, and concentrations of 17-OH-Pg and progesterone are also increased. Increased androgen synthesis in the ovary leads to the proliferation of theca and granulosa cells, causing a large number of small follicles to grow. The polycystic ovary has three times more antral follicles than a healthy ovary, while the number of primordial follicles is the same [15,16]. Androgen-induced follicular atresia is thought to involve excessive anti-Müllerian hormone (AMH), which inhibits follicular growth, dominant follicle selection, ovulation, and progesterone synthesis. Low progesterone levels increase pulsatile GnRH activity, further elevating LH and androgen production, while suppressing follicle-stimulating hormone (FSH), exacerbating follicular arrest and hormonal imbalance. The activity of FSH is also reduced by the action of inhibin B, estrogen, and local paracrine factors like follistatin and tumor necrosis factor, leading to an altered FSH/LH ratio [17,18]. This cyclical dysfunction underpins the clinical and morphological features of PCOS. Atypical functional ovarian hyperandrogenism (FOH), characterized by elevated testosterone, is rare but identifiable by suppression of adrenal androgen production via dexamethasone. FOH leads to an excess of small follicles, few of which progress to the preovulatory stage. This results in oligo-ovulation and polycystic ovarian morphology, often associated with elevated anti-Müllerian hormone (AMH) levels [19,20]. Functional adrenal hyperandrogenism (FAH) is detected in less than 10% of women with PCOS, with approximately 5% linked to congenital adrenal hyperplasia. The remaining cases of PCOS are mild, lack evidence of steroidogenic abnormalities, and are often associated with normal BMI, though they may also occur in women with obesity [21].

### 3.3. Obesity and PCOS

**Figure 2:**
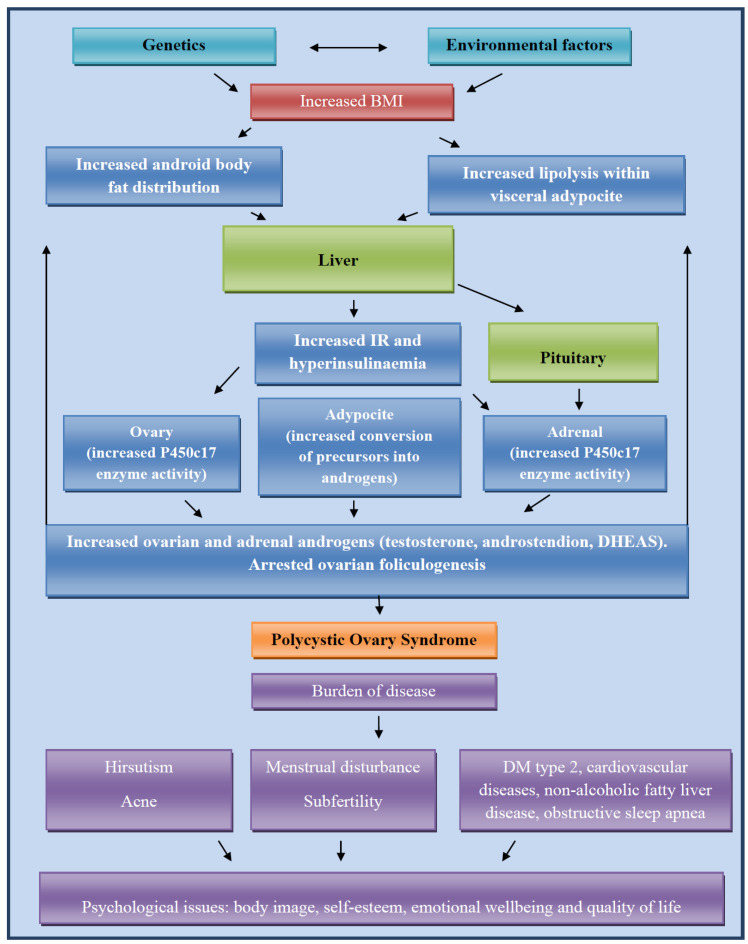
The prospective mechanism of PCOS and obesity pathogenesis.

Endocrine and ovulatory dysfunctions associated with obesity seem to be directly caused by its adverse effects on the hypothalamic–pituitary–ovarian axis (Figure 2). Table 1 shows findings of recent studies that demonstrated the connections between obesity, metabolic syndrome, and PCOS development. Obesity and PCOS have similar pathophysiological mechanisms, out of which insulin resistance and compensatory hyperinsulinemia, hyperandrogenism, and activation of the renin–angiotensin system from hyperaldosteronism are recognized long ago, while novel studies also suggest increased serum levels of 20-hydroxyeicosatetraenoic acid, apelin, and polygenic predisposition [6]. The latest studies show that IR and hyperandrogenism play the main role in pathogenesis, whereby IR is an inherent cause in PCOS independent of obesity [11,22]. In normal-weight patients with PCOS, the degree of IR is typically considerably lower than in obese patients with PCOS. IR was found to occur in 95% of obese and 75% of lean patients with PCOS. In patients with obesity, it is a result of intrinsic resistance as a part of the disease and extrinsic resistance due to obesity, while lean patients with PCOS have intrinsic IR [22,23,24]. Such results are supported by the fact that PCOS in lean patients usually presents during puberty, when a temporary rise in insulin-like growth factor-1 (IGF-1), growth hormone (GH) levels, and subsequent insulin levels and IR occurs. The steroidogenic ovaries and adrenal glands continue to have adequate sensitivity to the insulin actions, which cause clinical features of hyperandrogenism such as irregular menstrual cycle, acne, excessive hair growth, thinning hair, and darkened skin patches. Also, another effect of insulin is decreased synthesis of SHBG in the liver, which additionally results in elevated levels of free androgens [25,26,27]. Hyperandrogenism affects granulosa cell function and results in follicular dysplasia. Follicular dysplasia caused abnormal folliculogenesis, failed follicle selection, and anovulation, creating a specific morphology of the ovaries [27].

Unlike PCOS in lean patients, in patients with obesity, IR is usually a result of adiposity and decreased tissue sensitivity to insulin. That tight bond of overweight, obesity, and PCOS was proven by recent studies that showed that moderate weight reduction often resulted in clinically significant amelioration in the reproductive, hyperandrogenic, and metabolic characteristics of PCOS [23,26]. Obesity, especially visceral adiposity, which is typically found in obese patients with PCOS, increases the severity of insulin resistance and causes hyperinsulinemia, resulting in an increase in adipogenesis and lipolysis decrease in lipolysis. There is also a defect in the post-receptor phosphatidylinositol 3-kinase (PI3-K) insulin pathway, which interferes with the metabolic effects of insulin and makes the tissues develop resistance to the effects of insulin. Mitogen-activated protein kinase (MAP-K) pathway stays intact in patients with PCOS, with its dysmetabolic and steroidogenic implications that typify this condition [32].

According to previous research, all overweight and obese patients with PCOS have decreased levels of SHBG, increased testosterone and androstenedione, increased free androgen index, and all metabolic and reproductive features worsened. Obesity is associated with restrained ovulation and high LH serum levels that affect thecal cells and increase functional ovarian hyperandrogenism by upregulating ovarian androgen synthesis [28,33,34]. Menstrual irregularities are more common in obese patients with PCOS, as is the prevalence of IR and HOMA-IR, the prevalence of disrupted glucose tolerance, diabetes mellitus, and metabolic syndrome. Moreover, endometrial hyperplasia was considerably more common in the obese than in the non-obese patients with PCOS [29,35].

Women with PCOS are disposed to have visceral fat hypertrophy based on the androgen excess, linked to insulin resistance. On the other hand, impaired production of many adipocyte-derived substances (adipokines) is correlated with chronic low-grade inflammation and adds to the expression of IR. Abdominal obesity and insulin resistance stimulate ovarian and adrenal androgen synthesis, potentially leading to further abdominal obesity and inflammation, hence making a vicious cycle [6,36]. This inflammation has potentially a considerable impact on the regulation of ovarian functions as well as the disturbances in the levels of the inflammatory biomarkers, which are related to the ovarian dysfunction in PCOS [30].

In addition, literature data indicate that the non-alcoholic fatty liver disease is associated with lower free testosterone serum levels and free androgen index levels independently of insulin resistance and other factors. It is hypothesized that the hyperandrogenism possibly contributes to the progression and/or development of non-alcoholic fatty liver disease in PCOS [37].

### 3.4. Management of Obesity in PCOS

Taking into account that PCOS is a multisystem disease related to unhealthy living habits, genetics, and epigenetic factors, and one of the major reasons for infertility in the female population, it is no longer regarded as an illness of the sole ovary [31,38,39]. Consequently, the need for numerous therapeutic approaches and/or the combination of different therapeutic approaches for PCOS and related diseases has recently arisen. Different treatment approaches can be classified as non-pharmacological and pharmacological [39].

The non-pharmacological strategies for PCOS metabolic symptoms include the characteristics of lifestyle that can be changed and improved, such as different diets for weight loss (nutrition with reduced calories and glycemic index), introduction of regular mild everyday physical activity, and normalization of sleep patterns. Weight loss was linked to the metabolic, endocrine, reproductive, cardiovascular, and psychological characteristics improvement in overweight and obese patients with PCOS [40,41]. Thus, the low-calorie diets could be the most advantageous options for the correction of IR and improving the body composition of patients with PCOS [41,42]. The longer duration of the diet is associated with better health effects. The healthy diet compared to therapy with metformin was proven in some studies to be better for weight loss, while the impact on insulin regulation was comparable [43].

Another non-pharmacological lifestyle-modifying strategy for body composition modifications of patients with PCOS is the introduction of regular daily exercise. The physical training potentiates insulin sensitivity. It was found that the beneficial effects of the training are dependent more on the regularity of exercise than on intensity [44]. The physical training has the greatest impact on insulin resistance, cardiorespiratory fitness, and body composition of patients with PCOS. It has been reported that exercise lasting for a minimum of 120 min per week could provide favorable health outcomes for women with PCOS [31,44].

One more non-pharmacological approach for the treatment of obesity and health-related issues in patients with PCOS is bariatric surgery. Bariatric surgery is a widely used treatment for obesity, which can lead to a decrease not only in the BMI, but also in the serum total and free testosterone levels, ovarian volume, incidence of abnormal menstruation, and hirsutism of obese PCO patients [38]. On the other hand, in previous investigations, surgical treatment of obesity was not found to correlate with free androgen index, ovarian morphology, or homeostasis model assessment in patients with IR. The final BMI after bariatric surgery was confirmed as the parameter that has the strongest impact on the remission of PCOS [26].

It should be mentioned that indications for bariatric surgery differ between Western and Asian countries. The National Institutes of Health guidelines propose bariatric surgery for patients with a BMI ≥ 40 kg/m^2^ or BMI ≥ 35 kg/m^2^ who have severe comorbidities [31].

In Table 2, the comparison of the effectiveness of different therapeutic modalities in women with PCOS is presented.

### 3.5. Medications for PCOS in Patients with Obesity

Taking into account multiple etiological factors and pathways involved in the pathogenesis of PCOS and obesity, there are a couple of pharmacological strategies for the treatment of obesity in patients with PCOS. Although the lifestyle interventions represent a first-line treatment strategy for obesity in patients with PCOS, the significance of the anti-obesity medications for weight loss is considerable [27,32,33,34].

One of the most commonly used and efficient medications is metformin. Metformin is commonly used to treat PCOS as it successfully improves insulin sensitivity, reduces hyperinsulinemia, and lowers androgen levels [4]. There are multiple mechanisms of metformin action, such as the regulation of appetite, modulation of the gastrointestinal physiology and circadian rhythm, regulation of fat oxidation and storage in adipose tissue, skeletal muscles, and liver [27]. By activating AMP-activated protein kinase (AMPK), metformin decreases hepatic glucose production and enhances peripheral glucose uptake. It has also been shown to reduce testosterone and the free androgen index (FAI) while increasing sex hormone-binding globulin (SHBG), improving symptoms like hirsutism and acne. The therapy with metformin improves serum levels of FSH, LH, and low-density lipoprotein cholesterol (LDL) [32]. It has been reported that metformin reduces FSH levels in the human granulose cells by the downregulation of the FSH receptors and by lowering the FSH-induced phosphorylation of cyclic adenosine monophosphate response [33].

Additionally, metformin may contribute to weight loss and reduce the risk of type 2 diabetes and cardiovascular illness in patients with PCOS. Metformin suppresses appetite by the lactate-mediated metabolic acidosis, modulation of the gut–brain axis, increased production of the glucagon-like peptide 1 (GLP-1) and the anorectic hormone peptide YY, suppression of the hypothalamic AMPK, and decreases the leptin resistance in the hypothalamus [28,33,34]. Therefore, studies confirmed that the use of metformin leads to a significant reduction in waist circumference and improvement of the BMI. This also helps restore menstrual regularity and ovulatory function in obese patients with PCOS [33]. In spite of its advantages, several side effects of metformin use have been reported, primarily gastrointestinal disturbances (nausea, diarrhea, abdominal discomfort, bloating, and loss of appetite). These symptoms often occur at the beginning of treatment and may improve with gradual dose escalation and taking the medication with food. Prolonged therapy with metformin has been linked to vitamin B12 deficiency, which can lead to neuropathy, anemia, and cognitive impairments if left untreated [34]. Metformin suppresses the TLR4/IRF-7/NFκB signaling pathway in the endometrium of PCOS patients, which is overexpressed in the endometrium of patients with PCOS. It also inhibits the expression of the matrix metalloproteinase 2 (MMP-2) and matrix metalloproteinase 9 (MMP-9) and reduces progesterone receptor (PR) expression in the endometrium. Nevertheless, it has been reported that metformin use can lead to retrieving a lower number of oocytes during assisted reproduction as well as fertilized oocytes, but the live birth rate was similar in patients given metformin and placebo [28,29,35].

A group of drugs, called inositols, which include myoinositol and D-chiro-inositol, is involved in several biochemical processes within ovaries (insulin signaling and hormonal synthesis), causing a significant impact on oocyte maturation, fertilization, implantation, and post-implantation development [35]. Current studies have reported that treatment with myoinositols leads to the improvement in ovarian function and fertility, decreased intensity of hyperandrogenism and its symptoms like acne and hirsutism, positively affecting metabolic aspects and modulating a range of hormonal parameters incorporated in the reproductive axis functioning, including ovulation [29]. The clinical data suggest that myioinositol, D-chiro inositol, and their combination in a physiological ratio of 40:1 with or without adjuvant compounds can improve metabolic, hormonal, and reproductive aspects of PCOS [36]. Although the inositols are widely used in the treatment of lean patients with PCOS, their true mechanisms of action on carbohydrate and glucose metabolism are even now insufficiently comprehended. The beneficial effects of the inositols could be explained by several mechanisms such as enhancing insulin signaling as second messenger molecules in the energy metabolism phosphatidyl-inositol pathways of insulin-sensitive tissues including liver, muscle, and adipose tissue, which can cause improvement of insulin sensitivity, modulation of the activity of the steroidogenic enzymes which lower the androgen levels, anti-inflammatory effect, improvement in the mitochondrial function and supporting the follicular maturation and ovulation [30,37,38].

When combined inositol and metformin therapy was compared with metformin monotherapy, the improvement was noticed for the menstrual cycle regularity and the quality of life, while there were no differences in pregnancy rates, hormonal, and metabolic parameters [38]. Similar effects between metformin and myoinositol therapy were reported for obese patients with PCOS regarding the BMI, body composition, hormonal profile, metabolism of glucose and insulin, and adiponectin level [31].

Another compound, resveratrol, has positive effects in the treatment of PCOS. The resveratrol has antioxidant effects, decreases chronic inflammation and androgen levels, improves insulin sensitivity, and improves glucose and lipid metabolism [39]. Resveratrol is an effective therapy for patients with PCOS, and when compared to the placebo, it significantly reduced testosterone, LH, and DHEAS serum levels. The combination of resveratrol and myoinositol was found to be efficient in improving distorted endocrine parameters, metabolic indices, and stress burden in obese, oligo-anovulatory patients with PCOS [40].

Glucagon-like peptide-1 receptor agonists (GLP-1-RAs: liraglutide; semaglutide), besides the role in weight loss, can also impact the mechanisms involved in IR, leading to the increased expression of glucose transporters in insulin-dependent tissues, reduction in oxidative stress, decrease in inflammation, and modulation of the lipid metabolism [41]. GLP-1, an intestinal hormone, has significant physiological functions in glucose homeostasis regulation by stimulating the pancreatic production of insulin and inhibiting glucagon secretion [42]. The distribution of the GLP-1 receptors over the gastrointestinal, nervous, and reproductive systems supports the thesis that the GLP-1 integrates reproductive functions, metabolic system, nutrition, and energy homeostasis mechanisms [43]. GLP-1 impacts the ovarian function, especially the granulosa cells. Their administration was shown to produce attenuation of the ovarian granulosa cells’ apoptosis caused by PCOS in a concentration-dependent manner. Moreover, such effects are linked to changes in the phosphorylation sites of forkhead box protein O1 (FoxO1) and a negative regulation of cell survival [44]. GLP-1 also suppressed the FSH-induced synthesis of progesterone through the inhibition of the progestogenic factors and enzymes [48]. The treatment with the GLP-1 agonist can decrease the intensity of inflammation and fibrosis both in the ovary and endometrium in animal models. This was an important finding because one of the most prevalent metabolic impairments related to obesity in patients with PCOS is inflammation [49,50]. GLP-1 exerts an anorectic effect through the hunger-satiety centre located in the hypothalamus, and by postponing gastric emptying. The therapy with GLP-1 agonists could improve fertility either by increasing LH surge in hypothalamus-pituitary inhibition caused by obesity-related estrogen surplus or by reducing high LH levels accompanying hyperinsulinemia [50]. When compared with the metformin monotherapy, the combination of the GLP-1 agonist and metformin was found to cause better reduction in weight, waist circumference, and BMI. In addition, the levels of fasting glucose, oral glucose tolerance test (OGTT) 2 h glucose, and OGTT 2 h insulin were considerably lower in patients who received both GLP-1 agonist and metformin when compared with the metformin only therapy [51]. Moreover, the combination of metformin and GLP-1 agonists improved serum levels of estradiol, luteinizing hormone, follicle-stimulating hormone, and progesterone as well as parameters of hyperandrogenemia, including total testosterone, sex hormone binding globulin, and free androgen index, while metformin monotherapy only improved estradiol and sex hormone binding globulin rates and free androgen index [52]. One meta-analysis showed that the GLP-1 receptor agonists are more effective in the insulin sensitivity improvement as well as in reducing BMI and waist circumference compared to metformin. On the other hand, GLP-1 receptor agonists may be linked to more frequent occurrence of nausea and headache than metformin, but no noteworthy differences in the occurrence of other adverse effects were registered so far [45,46,53,54]. Another meta-analysis reported that the therapy with GLP-1 agonists in comparison to metformin caused increased pregnancy rates, greater ovulation rate, decreased body mass index, and improved IR [46]. Metformin combined with thiazolidinediones could be particularly effective in promoting the recovery of menstruation, but thiazolidinediones are found to be second-rate to metformin for lowering BMI [45]. Mutual therapy with metformin and GLP-1 receptor agonists brings the additional benefit of improving fasting glucose when compared with GLP-1 receptor agonist monotherapy [45,46,54]. The preconceptional intervention with low doses of the GLP-1 agonists in combination with the metformin monotherapy was found to be linked with higher pregnancy rates per embryo transfer as well as cumulative pregnancy rates in infertile obese patients with PCOS [46]. In Table 3, the comparison of the effectiveness of different pharmacological therapies in women with PCOS is presented.

### 3.6. Differences in Treatment Between Obese and Lean Patients with PCOS

In obese PCOS patients with a BMI over 30, there are opportunities to include GLP-1 agonists as an additional therapy [59,60]. First, it is necessary to assess the risk of type II diabetes mellitus, fatty liver disease, and metabolic syndrome presence [60]. If patients are predisposed to these conditions, it is preferable to include GLP-1 agonists in the initial stages of treatment [60]. Conversely, the use of GLP-1 agonists is not recommended in lean patients with PCOS, so as additional weight loss may additionally block hypthalamo–hypophyseal axis, which then may presents a major challenge both for diagnosis and further treatment [32]. Sudden and significant weight loss can worsen pre-existing amenorrhea and PCOS overall, so in such cases, the use of inositol is more advisable [61]. A similar effect may be seen with long-term use of contraceptives, whereas antiandrogens may improve the clinical presentation of hyperandrogenism [62]. Advised therapeutic approach is presented in Table 4.

## 4. Discussion

Obesity is a widespread problem in women with PCOS, and therefore, weight loss is the most important step in the management of obese patients with PCOS [63]. In addition, obesity represents a risk factor for the development of the metabolic syndrome, dyslipidemia, IR, diabetes mellitus, cardiovascular diseases, subfertility and infertility, the entities closely related to PCOS [55]. The real challenge in the treatment of obesity in patients with PCOS is the strong physiological compensatory mechanisms that increase the desire for feeding to restore energy balance, in contrast to the weight loss via physical activity, pharmacological, or surgical management [55,56].

Recent investigations have proven that although all patients with PCOS, regardless of BMI, share the same core characteristics (hormonal imbalances and anovulation), metabolic profiles and clinical presentations differ between obese and lean PCO patients, and therefore they require different treatment approaches. Therefore, the most appropriate PCOS division would be into reproductive and metabolic types, as already suggested [47].

PCOS metabolic types of patients generally present with more severe clinical and biochemical manifestations such as higher hyperinsulinemia prevalence, elevated fasting insulin and IR, dyslipidemia with elevated triglyceride and LDL levels, menstrual irregularities, and hyperandrogenic symptoms like hirsutism and acne. These patients also more frequently have impaired vascular smooth muscle function compared to their lean counterparts [57,64].

On the other hand, PCOS reproductive types of patients can either present with key metabolic disturbances or may have normal insulin levels and no clinically relevant insulin resistance, as well as decreased HDL cholesterol levels (mostly A and B phenotypes). These patients frequently have elevated leptin and lower adiponectin and vitamin D serum levels, which are associated with IR [65].

Moreover, lean and obese patients with PCOS naturally differ in body composition and adipose tissue distribution. Although both patient groups have disproportionately higher visceral adiposity, android (central) obesity shows a higher prevalence in metabolic types of patients with PCOS [66]. These patients tend to have more prominent clinical expression of hyperandrogenism, including a higher hirsutism score (moderate or severe) and severe acne than lean patients. Studies have shown that menstrual irregularity may also be more prevalent in obese patients with PCOS compared to their lean counterparts. However, hormonal profiles may differ subtly between these two patient groups. While both of them may present elevated LH/FSH ratios, lean women more often have also elevated DHEAS levels [67,68].

As for the reproductive outcomes, metabolic types of patients with PCOS more commonly require higher doses of ovulation induction agents with poorer responses during IVF, while reproductive types of patients with PCOS have better reproductive outcomes as they mostly maintain ovulatory capacity with favorable responses even on the first-line ovulation induction therapy [69].

Based on the findings from recent studies, it is evident that patients with different PCOS profiles may benefit from customized therapeutic approaches [65,66,67,68,69]. Weight loss is often a primary treatment goal for obese patients with PCOS, whereas lean patients with PCOS may not require weight loss, but should focus on maintaining their weight. Lifestyle modifications, including dietary changes and regular exercise, are important for managing PCOS symptoms in both groups of patients. Still, lean patients have a better response to lifestyle modifications than obese patients with PCOS [69].

Different pharmacological approaches are useful for the treatment and weight loss in women with PCOS [58,70]. Most studies highlight the effectiveness of combined therapies, particularly those incorporating metformin and GLP-1 receptor agonists, like liraglutide and exenatide, for the management of both metabolic and reproductive issues linked to PCOS. Although the metformin base therapy has long been established as the first-line pharmacological therapy for PCOS, the evidence suggests that its efficacy is significantly improved when combined with GLP-1 receptor agonists [15,35]. The significant improvements observed in reproductive outcomes and androgen levels during this combined therapy are important for patients presenting with symptoms such as infertility or menstrual irregularities. This approach improves not only insulin sensitivity but also contributes to weight loss and the restoration of ovulation. The findings of meta-analyses indicate that patients with PCOS who are overweight or obese, who have problems conceiving, may experience substantial benefits from this combined therapy [35,71]. These results emphasize the need to thoroughly assess patients’ body weight and composition before selecting a specific treatment regimen. For patients with PCOS who are not overweight or who have contraindications for GLP-1 agonists, there are alternatives such as myoinositol, resveratrol, or their combination [71]. The available data suggest that myoinositol offers benefits for menstrual regularity and overall quality of life. On the other hand, the use of resveratrol leads to a significant decrease in testosterone serum levels and improves the hormonal milieu, making it a suitable adjunct therapy for patients with hyperandrogenism [35]. Furthermore, the combination of metformin with thiazolidinediones is a promising novel therapy for restoring menstrual regularity, but this combination may be less effective in obesity management when compared to the metformin-GLP-1 combination [35,71].

Regarding all the above mentioned, it can be seen that future clinical research should investigate the optimal and individualized utilization of GLP-1 agonists along with other therapies for obese patients with PCOS.

## 5. Conclusions

PCOS, as a multifactorial chronic disease, can cause heterogeneous metabolic, physical, and psychological disorders, as well as infertility in both obese and non-obese patients. It is a time-consuming condition in terms of diagnostics and treatment. Currently, the first line treatment for PCOS is a lifestyle-modifying strategy with a specific diet and regular daily mild physical activity for body weight and composition management. The second line of therapy is pharmacological treatment. The bariatric surgical intervention is only indicated for the treatment of PCOS patients with and a BMI higher than 35–40 kg/m^2^ with comorbidities.

PCOS patients with insulin resistance and obesity would mostly benefit from the combined treatment with metformin and GLP-1 receptor agonists. Conversely, those with normal BMI, milder symptoms, or contraindications to this therapy may find relief with inositols, like myoinositol, with the addition of resveratrol. Individualized treatment strategies according to patients’ BMI, WH ratio, and metabolic profiles may be the optimal choice to fulfill the clinical requirements of patients with PCOS and optimize both fertility and long-term health outcomes.

## Figures and Tables

**Table 1:** The association of obesity, metabolic syndrome and PCOS development.

Studies Comparing Obese and Non-Obese Patients	Population	Main Results and Conclusions
Gholinezhad M et al. (2018) cross-sectional study [27]	27 normal weight (18 < BMI < 25) and 85 overweight/obese (BMI ≥ 25) patients aged 18 and 35 underwent clinical measures of HOMA as insulin resistance and QUICKI as insulin sensitivity tools	BMI was significantly positively correlated with insulin resistance (*p* < 0.001) and negatively with insulin sensitivity (*p* < 0.001). BMI showed stronger reverse relationship with SHBG (*p* < 0.001). In the overweight/obese group, 91.7% of the women showed insulin resistance (HOMA > 3.15) vs. 8.3% in the normal weight group (*p* < 0.001). Low insulin sensitivity (QUICKI < 0.34) had 17.6% of the lean women and 82.4% of the overweight/obese patients (*p* < 0.001).
Stepto NK et al. (2013) cross-sectional study [28]	20 overweight and 20 lean PCOS patients (Rotterdam criteria)	Women with PCOS had more IR than BMI-matched controls (main effect for BMI and PCOS; *p* < 0.001). IR was present in 75% of lean PCOS, 62% of overweight controls and 95% of overweight PCOS. Lean controls (mean + SD; GIR 339 + 76 mg) were less IR than lean PCOS (270 + 66 mg), overweight controls (264 + 66 mg) and overweight PCOS (175 + 96 mg). The negative relationship between BMI and IR reflected by GIR was more marked in PCOS (*p* < 0.0001) than controls (*p* < 0.01).
Bailey AP et al. (2014) retrospective cohort study [29]	79 women with clinically documented diagnosis of PCOS by Rotterdam criteria undergoing IVF	Women with obesity and PCOS had 69% lower odds of clinical pregnancy per started cycle (OR 0.31; 95% CI, 0.11–0.86; *p* = 0.02) and 77% lower odds per embryo transfer (OR, 0.23; 95% CI, −0.08 to 0.68; *p* = 0.008) compared with lean women with PCOS. Among women with obesity and PCOS, the odds of live birth were 71% lower per started cycle (OR, 0.29; 95% CI, 0.10–0.84; *p* = 0.02) and 77% lower per embryo transfer (OR, 0.23; 95% CI, 0.07–0.71; *p* = 0.01) compared with lean women with PCOS. Ovarian hyperstimulation syndrome odds were decreased with increasing BMI among PCOS patients: 19.6% in lean, 10.5% overweight and 3.2% obese women.
Johnson JE et al. (2023) meta-analysis [30]	10 relevant studies were identified and included (12,248 patients with PCOS and 54,120 controls)	Women with PCOS had a significantly increased odds of developing endometrial cancer as compared to those without PCOS [OR, 4.07; 95% confidence interval (CI), 2.13–7.78; *p* < 0.0001]. When postmenopausal subjects (age > 54 years) were excluded from the meta-analysis, the odds increased further (OR, 5.14; 95% CI, 3.22–8.21; *p* < 0.00001). Patients with PCOS are up to 5 times more likely to develop endometrial cancer compared to those without PCOS.
Hong SH et al. (2023) cross sectional observational study [31]	667 patients with PCOS and 289 women with regular menstrual cycles as control	The prevalence of NAFLD in women with PCOS evaluated by LFS, FLI, and HIS were 19.9, 10.3, and 32.2%, respectively. In the control group, the incidence was 2.1, 0.7, and 4.2%, respectively. Both FT and FAI levels showed significant association with increased NAFLD-related indices, after adjusting for insulin resistance and other factors (LFS (OR 3.18 (95% CI 1.53–6.63) in FT; 1.12 (1.04–1.22) in FAI), FLI (OR 2.68 (95% CI 1.43–5.03) in FT; 1.13 (1.06–1.20) in FAI), and HSI (OR 3.29 (95% CI 2.08–5.21) in FT; 1.5 (1.09–1.21) in FAI). TT did not exhibit association with any NAFLD index.

**Table 2:** The non-pharmacological and pharmacological therapy for obese patients with PCOS.

Studies	Population	Main Results and Conclusions
Shang Y et al. (2020) Meta-analysis [40]	19 trials with 1193 patients	Diet leads to more pronounced improvement in the homeostasis model assessment of IR, fasting insulin, fasting plasma glucose, BMI, weight, and waist circumference in PCOS patients compared to the metformin group.
Porchia LM et al. (2020) Meta-analysis [41]	25 studies with 486 patients	Diet leads to the significant improvement in the IR in women with PCOS.
Patten TK et al. (2020) Meta-analysis [44]	19 studies with 777 patients	The physical activity leads to the small reductions in HOMA-IR and waist circumference.
Chen M et al. (2024) Meta-analysis [44]	9 studies with 1330 patients	Bariatric surgery lowers menstrual irregularity, BMI, ovarian volume hypertrichosis and free testosterone levels in women with obesity and PCOS.
Guan Y et al. (2020) Meta-analysis [45]	12 studies with 683 patients	The metformin leads to the significant decrease in the BMI, waist circumference, LDL, FSH, LH, and testosterone levels in women with obesity and PCOS.
Greff D et al. (2023) Meta-analysis [46]	26 studies with 1691 patients	The inositol leads to the significant decrease in free testosterone, total testosterone, androsenedione, glucose, AUC insulin as well as the BMI and increase in the SHBG compared to the placebo.
Ali Fadlalmola H et al. (2023) Meta-analysis [47]	4 studies with 218 patients	The resveratrol significantly decreases the LH, testosterone and DHEAS levels in women with PCOS compared to the placebo in women with PCOS.

**Table 3:** The effectiveness of different pharmacological therapies for PCOS.

Studies	Population	Main Results and Conclusions
Nazirudeen R et al. (2023) Randomized controlled trial [55]	53 patients (27 treated with metformin 1500 mg/day and 26 treated with metformin 1500 mg/day and myoinositol 4 g/day)	In comparison to the metfomin monoterapy, combination of the metformin and mioinositol leads to the significant improvement in the menstrual regularity. There is no significant difference in the anthropometric parameters, modified Ferriman Gallwey score, global acne score, Fasting insulin, HOMA-IR, fasting lipid profile, serum testosterone, SHBG, LH, FSH, AMH, and pelvic ultrasound to assess ovarian volume.
Soldat Stankovic V et al. (2022) Randomized controlled trial [56]	66 patients treated with metformin 1500 mg/day and myoinositol 4 g/day	There is no difference in total cholesterol, HDL, LDL cholesterol and triglycerdies between treatment with metformin and myoinositol. Metformin treatment significantly reduces the BMI, waist circumference, Ferriman Gallwey score testosterone and FAI.
Hassan S et al. (2023) Randomized controlled trial [57]	110 patients (55 treated with metformin 1000 mg/day + pioglitazone 30 mg/day and 55 treated with myoinositol 2000 mg/day + resveratrol 2000 mg/day)	The treatment with the combination of the myoinositol and resveratrol is more efficient in the lower of the testosterone, LH, FSH, ovarian volume, BMI, WH ratio and Ferrimen-Gllwey score when compare to the treatment with the combination of the metformin and pioglitazone.
Xing C et al. (2020) Meta-analysis [58]	14 studies with 619 patients	The combination of the GLP-1 receptor agonists and metformin is more effective in decreasing of the free testosterone, androstenedine, fasting blood glucose when compare to the metformin monoterapy while the combination of the metformin and GLP-1 receptor agonists is more effective in increasing SHBG when compared to the GLP-1 RA monotherapy.

**Table 4:** Differences in treatment between obese and lean patients with PCOS.

Obese Patients with PCOS	Lean Patients with PCOS
1. weight reduction and lifestyle changes	1. inositol; resveratrol
2. metformin (depending on metabolic status)	2. hormonal contraception
3. inositol; resveratrol(with or without metformin)	3. antiandrogens
4. GLP-1 receptor agonists liraglutide for obesity treatment of patients with no metabolic disorders semaglutide for obesity treatment of patients with DM type II	

## Data Availability

Data are contained within this article.
